# Curantur *vs.* Curentur

**Published:** 1896-12

**Authors:** E. L. Close

**Affiliations:** Secretary


### CURANTUR *vs.* CURENTUR.

(Proceedings of the Brooklyn Hahnemannian Union.)

At the regular meeting of the Union, held May 30th, 1896, Dr. Fincke presented a paper on "Curantur *vs.* Curentur," upholding the use of the former term. Hahnemann's first use of a formula expressing the homœopathic law was in his "Essay on a New Principle," published in *Hufeland's Journal,* in 1796. It was there written simply *similia similibus,* as it was also in an article in the same journal in 1805. The copula does not appear until 1819, in the introduction to *The Organon,* second edition, page 29, where it is printed *curentur.* The same spelling is repeated in the third, fourth, and fifth editions, but the formula appears only in the introduction. Nowhere in the text of his *Organon,* in all its five editions, can be found the Latin sentence with the "*curentur*" when he speaks of the homœopathic law. Hence the writing of "*curentur*" is not binding. Hahnemann was at first satisfied with the simple motto *similia similibus*, and this might even now be desirable, as intimating the wider range of the Hahnemannian principle, since nothing in the world ever moves or has its being except on this universal principle of assimilation, underlying the universal principle of gravitation. Hahnemann preferred *curentur*, because it included with the acknowlment of the philosophical principle the rule which enjoined the physician to attend to the sick according to his newly proclaimed principle. But as a broad declaration of principle, as against an imperative admonition to apply the principle, *curantur* is preferable, and that expression has come generally into use.

Dr. Baylies suggested that both expressions were valuable. "Let similiars be cured by similiars" is the result of experience.

Dr. Fincke said that the subject was brought up in the discussion in regard to the inscription on the monument to be erected to Hahnemann in Washington. If we enunciate a law it must be positive. Newton did not say, "Let action and reaction be equal." The law is declared with no doubt, and it is safer to say "curantur."

Dr. Baylies thought "curentur" might be distinguished as precept, and "curantur" as law.

Dr. Close asked for a literal translation of "similia similibus," and Dr. Fincke answered, simply, "Likes by likes," or, Dr. Baylies added, "Likes with likes."

Dr. Fincke said it was the talk about the relation of *simile* with *equal* that gave great trouble in Germany, some wishing to use the expression "equal with equal;" but Hahnemann protested that they were only similiar, and so it remained. *Idem* was not used because two things cannot be the same.

Dr. Close noted the fact that the matter is now receiving attention because of ideas Dr. Fincke has brought forward, the idea of a series of similes being a revelation, throwing light on the different degrees of success with the remedies. If our cures depended upon our always finding the equal, we should be in a box, but while the similiar helps and ameliorates, we can keep on striving for the highest similiar until we are expert enough to find the simillimum, which is the equal.

Dr. Fincke said, Hahnemann never talks about what is similiar. There ought to be a definition. If this is adopted, that *the simillimum is the equal*, it is put upon a firm basis. If you can find in a patient a single complete symptom, fully and accurately expressed, and can then find that symptom in the *Materia Medica*, you have found the curative remedy. If in a group of twenty or thirty symptoms we try to find the equal of the most prominent symptom, we will get near the simillimum, and always do something for the best. Nature will do for us what we cannot do for ourselves.

Bœnninghausen tells of a cattle disease in his neighborhood, where the animal got stiff, refused to eat, and died in a few days. He had two valuable cows, and put up two powders, one of Puls. and one of Nux. For one of them, that was taken ill, the Puls. was given, and in the morning the cow was well, which made a great sensation. To the other cow, which was now ill, the Nux was given, and that cow also promptly recovered. Which was similiar? As in a picture, what different ideas people have of the similiarity; one finds an exact likeness where the other cannot see it at all.

Dr. Alice Campbell objected that we are not allowed to use our own judgment where law is concerned.

Dr. Fincke said it depends upon the analysis of the picture.

Dr. Close thought there was room for judgment in the method of applying the law, but no room for judgment in the necessity to apply the law.

Dr. Fincke gave as an illustration of this the case of a gushing hemorrhage in confinement, where Ipecac. helped, but when the patient complained of heaviness of the eyes, and a yellow saddle was noticed across the nose, Sepia was given, which cured. So unusual symptoms, apparently trifling, are sometimes the most important, and there individual judgment is needed.

Dr. Baylies told of a case of erysipelas of the face, beginning on the nose. The patient had formerly had liver spots on both cheeks. There was itching and burning, with a desire to apply something soothing. Sulphur was given erroneously, which increased the itching, but Sepia$^{45m}$ helped the itching in a few minutes, and daily improvement followed.

Dr. Alice Campbell thought that, according to these statements, the selection of the simillimum was not based upon vital symptoms, adding that Dr. Wells, after repeated failures in one case, made a prescription on a wart up under the hair with success. This wart, like the saddle across the nose, was the most important symptom.

Dr. Fincke suggested that it was like a large house with a little door and a tiny key which opens to the whole house.

Dr. Close said that a case of hemorrhage, for which the allopath uses local measures, needs thoroughness of examination. The homœopath looks further to the predisposing causes. The saddle on the nose in this case was the last link in a chain of conditions running back many years. He forms a deeper philosophy of that case, because he goes into the history of the case, and includes pre-existing as well as present symptoms.

Dr. Campbell said that we naturally suppose such a condition to be an advanced one, and wish to attend to the latest symptoms.

Dr. Close referred again to the series of similars. Possibly some other remedy would have helped, as did the Ipecac., but Sepia proved to be the simillimum.

Dr. Campbell gave the saying of Dunham : "In chronic conditions get the symptoms of an acute attack, which will give the simillimum."

Dr. Close expressed gratitude for the host of workers before us who have found the key-notes of the remedies, thus facilitating our work.

Dr. Fincke said the pathological explanation of the saddle on the nose has never been given. We do not need to know, only to take the fact. Thus we are always in advance of science. They may not find the cause in a thousand years.

Dr. Baylies spoke of spots in other parts of the body, the back and abdomen, as also indicating Sepia.

Dr. Close mentioned other spots which are an absence of pigment, such as white spots left in tan, for which no cause is known.

Dr. John Campbell said that dermatologists refer them to an absence of pigment in one place and excess in others.

Dr. Baylies related the case of a lady, who is sometimes attacked in the night with a hacking cough, lachrymation, coryza; cough worse on lying down; wants to cover head. Nat-carb. generally stopped it immediately. But a few nights ago it did not help, and the next evening she had a sudden attack of earache; throbbing in the right ear, soreness of the external ear. Pulsatilla$^{40m}$ helped for a few minutes only. Then the pain increased, and extended to the left ear. Puls. millionth relieved for half an hour. The soreness was better, but the throbbing was worse, like an internal pumping, with constriction in the throat. Glonoine$^{45m}$ soon relieved. Before taking it there was a discharge from both ears; increased in the right. She was very deaf, and in a few days went to see Dr. Schenck, who found interstitial hemorrhage in the tympani of both ears.

Dr. Close said Rumex and Psorinum both want the head covered, but Dr. Baylies said Psorinum wants to lie down.

Dr. Campbell remarked that Puls. couldn't work beyond its own sphere, relieving the external ear. She also referred to her recent illness, which was an exemplification of Puls. with the nervous symptoms and pain in the bones of the face.

Dr. John Campbell brought up the statement made by a Swedenborgian minister that remedies acted on the mind through the physical being only, and Dr. Baylies asked, How can we bring the medicinal force to bear upon the mind except through a physical medium?

Dr. Campbell said that Hahnemann's statement was that spirit-force acts upon spirit, which Dr. Baylies corrected as "spirit-like force."

Dr. Fincke said force must act upon force. We cannot have force without it is attached to matter, the vehicle.

Dr. Baylies said spirit can act upon spirit.

Dr. Fincke asked: "Tell me what spirit is. *Matter is a vehicle for force*, but what is spirit? I have no idea."

Dr. Baylies asked: "Do you believe your spirit can live apart from your body?" to which Dr. Fincke answered: "I am sure it will live after I die."

Dr. Baylies said: "Then the spirit can live apart from the body."

Dr. Fincke thought that was beyond human ken, unless we speculate and fancy, which is no use. What matter is I won't break my head about. It is nothing by itself. Force doesn't come from matter, because it is inert.

Dr. Baylies said the form of the matter changes the form of the force. Different kinds of force comes from different forms of matter. There must be something peculiar in the different forms.

Dr. Fincke answered: The forces are given. Who put the Silicea force in the grain of sand? It was given by the great Almighty Force, and they always stay their own; that's the wonder. There is no change when the potency is run up.

Dr. Campbell asked if force shapes matter or matter shapes force?

Dr. Fincke answered: Matter is inert, and cannot shape or produce or change force.

Dr. Baylies said: What shapes matter? Each specific force gives shape to the matter which contains it.

The meeting then adjonrned.

Respectfully submitted,

E. L. Close, *Secretary.*

